# Learning Health Systems for Chronic Disease: Embedded Artificial Intelligence Tools That Continuously Improve Care

**DOI:** 10.5888/pcd23.260103

**Published:** 2026-10-01

**Authors:** Anand Gourishankar, Sophia Z. Shalhout

**Affiliations:** 1 Department of Pediatrics (Hospital Medicine), Children’s National Hospital, Washington, District of Columbia; 2 George Washington University School of Medicine and Health Sciences, Washington, District of Columbia; 3 Division of Surgical Oncology, Department of Otolaryngology–Head and Neck Surgery, Mike Toth Head and Neck Cancer Research Center, Mass Eye and Ear, Mass General Brigham, Boston, Massachusetts; 4 Department of Otolaryngology–Head and Neck Surgery, Harvard Medical School, Boston, Massachusetts; 5 Computational and AI Services Program, Mass Eye and Ear, Mass General Brigham, Boston, Massachusetts

Chronic disease care demands sustained control and ongoing adaptation, making it distinct from acute care (1). In this type of care environment, populations change, workflows shift, health insurance coverage fluctuates, environmental conditions vary, and social context evolves; therefore, to be useful, a predictive artificial intelligence (AI) tool must remain reliable despite such changes. Further, the tool must remain clinically useful and must ensure that social determinants of health are accounted for (2). The AI tool therefore must be designed for ongoing adaptation in real-world settings, then monitored and updated. As health care data, patient populations, and social determinants of health evolve, AI models are vulnerable to performance degradation and bias. Addressing this challenge requires a comprehensive approach centered on ongoing monitoring, drift management, and governance to ensure sustained effectiveness, fairness, and clinical relevance.

A learning health system (LHS) offers a useful framework for addressing this challenge (3). An LHS embeds AI tools within an ongoing cycle of data capture, interpretation, action, and re-evaluation. In this framework, AI tools help identify emerging patterns and convert real-world data into knowledge that supports organizational learning. Data and outcomes from frontline care provide feedback to system leaders and learning processes, informing decisions about care delivery, resource allocation, and model management. These decisions then generate feedback to frontline care through revised workflows, updated clinical guidance, targeted interventions, and model refinements. In this way, predictive AI tools support bidirectional feedback between frontline care and system-level decision-making within a structured framework of integration and governance (4). We argue that when embedded within an LHS, the value of predictive AI tools in chronic disease care lies not only in their capacity to predict but in their capacity to continuously monitor, detect drift, and bring about adaptation.

## AI’s Role in a Learning Health System

An LHS does not treat AI tools as a stand-alone product; it treats AI as 1 component within a broader cycle of data capture, interpretation, action, and re-evaluation. In this context, the contribution of the AI tools is not limited to prediction. They help transform large, heterogeneous, and rapidly changing data streams into usable signals for clinicians, care teams, and health systems. Conceptual work in this area describes AI-enabled LHSs as bidirectional learning systems in which patient-level data generate analytic insight. Those insights inform organizational decisions, and updated guidance is then returned to the point of care through structured workflows and governance. In that sense, AI can accelerate learning across the micro-to-macro continuum, but only when paired with organizational processes, including interpretation, implementation, and reassessment over time (4).

This framing underscores that predictive AI tools are not themselves learning health systems but can operate as components within them. Although AI tools generate predictions and insights, LHSs provide the structures for monitoring, evaluation, feedback, governance, and continuous adaptation required to ensure those insights improve care over time. These tools can support the LHS function by detecting patterns that would be difficult to identify through conventional review alone (eg, emerging disease risk trajectories, changes in treatment response, variations in health care use, and early signals of adverse outcomes). Thus, an AI-supported LHS can better support dynamic, multimodal, and widely distributed clinical, behavioral, and contextual domains by integrating diverse data sources, identifying emerging trends, and enabling timely adaptation of care and system processes.

Predictive AI tools can translate those patterns into operationally meaningful outputs, such as risk flags, outreach lists, or decision support prompts embedded in care delivery. Additionally, these AI tools enable feedback from outcomes back into system learning, so that model outputs are evaluated not only for technical performance but also to improve clinical, operational, and patient-centered outcomes in practice. Recently developed frameworks emphasize that system learning depends on workflow integration, data quality, governance, and trust; without these, AI may generate predictions, but it does not reliably generate system learning (5,6).

## Pediatric Asthma: Using AI Tools Within an LHS

Pediatric asthma is a promising area to demonstrate why AI tools should operate within an LHS. Because the risk of asthma changes with seasonality, medication access, environmental exposures, and social context, AI tools can help detect emerging patterns in longitudinal data, such as gaps in prescription refills, recurrent emergency visits, rising use of rescue medication, or geographic clustering of exacerbations (4,7). The LHS then supplies the operational framework to act on those signals through proactive outreach, medication review, inhaler-technique reinforcement, expedited follow-up, and environmental or social needs assessment, while monitoring whether these responses improve outcomes and reduce health care disparities over time. Recent work in pediatric asthma LHSs supports the value of this coordinated, cross-setting approach (7). In the asthma LHS described by Beck et al, shared dashboards and multidisciplinary action huddles already provide the organizational scaffold for coordinated learning and response. AI tools, for example, could add value by extending this infrastructure from descriptive pattern recognition to automated detection of higher-risk trajectories. These trajectories may include longitudinal and multimodal data streams. Therefore, making AI tools a component of an LHS helps teams to prioritize which patients, neighborhoods, or seasonal patterns warrant earlier intervention. A recent review on the use of AI in asthma care highlights the role of AI within an LHS, particularly its ability to synthesize fragmented longitudinal multimodal data into workflow-concordant point-of-care decision support. Findings showed that AI also enabled ongoing monitoring, fairness assessment, and life cycle governance over time (8).

## Operational Pillars: Monitoring, Drift, and Governance

For AI tools to function within an LHS, health systems should establish a longitudinal monitoring dashboard. This dashboard needs to be reviewed at regular intervals, and the review should assess 3 pillars: monitoring, drift (calibration over time), and governance. Calibration should assess whether predicted risk remains aligned with observed outcomes as care patterns and populations change. Monitoring should examine performance across clinically and socially relevant strata, including geography and other markers of inequity, to ascertain whether average model performance obscures differential error or worsening disparities. Outcome trends should extend beyond technical metrics to include measures such as disease control, avoidable use of acute care, and follow-up completion. This kind of dashboard allows AI tools to be evaluated as part of care delivery rather than as a 1-time technical product (2,5).

Monitoring becomes useful only when drift triggers and response pathways are defined in advance. At a minimum, systems should specify 2 or 3 conditions that automatically prompt review, such as a sustained decline in calibration, worsening subgroup performance, or divergence between predicted risk and observed clinical outcomes after a workflow or population change. A standing governance group should then be responsible for reviewing these signals and coordinating corrective action. This group should include clinical leadership, quality improvement experts, technical experts, and those responsible for oversight of equity. The group should oversee whether recalibration, workflow redesign, targeted outreach, or temporary suspension of model-guided decisions is warranted. AI tools add value when the system can learn from its use over time and respond when performance shifts (4,9).

## A Practical Path Forward

For chronic disease prevention, the central challenge is not whether AI tools can generate accurate predictions at a single point in time, but whether those predictions remain useful, equitable, and actionable as clinical and social conditions change. A path to improved care involves the use of LHS with embedded AI tools. The AI-supported LHS can monitor performance longitudinally, detect drift, identify inequities, and support timely corrective action (10). In this model, the value of AI is in the system’s capacity to learn from use, adapt workflows, and refine care over time. Framed this way, AI tools can contribute meaningfully to chronic disease prevention only when embedded within organizational structures that support continuous evaluation, governance, and improvement.
